# The evolution of applied field epidemiology training in Canada: The last fifty years, and a vision for the future

**DOI:** 10.14745/ccdr.v52i78a02

**Published:** 2026-07-30

**Authors:** Benjamin Hetman, Esmé Lanktree, Carolyn Dohoo, Joanne Stares, Marc-André Bélair, Lisa Jensen

**Affiliations:** 1 Training and Development Unit, Centre for Emergency Preparedness, Public Health Agency of Canada, Ottawa, ON

**Keywords:** field epidemiology, training, adult learning, applied public health training

## Abstract

In Canada, successive crises (e.g., severe acute respiratory syndrome [SARS] in 2003, H1N1 in 2009, COVID-19 in 2019) have not only tested the response capacity of the public health workforce, but reshaped how field epidemiologists are trained. Over five decades, federal applied epidemiology training in Canada has evolved from a small, in-person program focused on outbreak investigation to a multidisciplinary, nationally coordinated training ecosystem integrating emergency management, health equity, climate responsiveness, and technological fluency. This commentary reflects on that evolution and considers the future of the Field Epidemiology Training Program in an era of intersecting and accelerating public health needs.

## Introduction

Looking back: The evolution of federal applied epidemiology training in Canada in response to public health emergencies

Applied epidemiology training for public health practitioners has been a core part of the Canadian federal public service for over 50 years. Beginning in the early 1970s, Epidemiology in Action (EIA) was established as an instructional model for applied epidemiology training in surveillance and outbreak investigation in Canada. The country’s Field Epidemiology Training Program (FETP) was formally established in 1975 and named the Canadian Field Epidemiology Program (CFEP). Since its founding, EIA training has formed the basis of classroom instruction and has since helped to shape 50 cohorts of Canadian field epidemiologists (1).

Major public health emergencies have informed the priorities of CFEP training throughout the program’s history. For example, the 2003 severe acute respiratory syndrome (SARS) outbreak exposed critical weaknesses in national coordination and surge capacity (2) and highlighted the importance of field epidemiology training in Canada. The subsequent creation of the Public Health Agency of Canada (PHAC) in 2004 formalized a federal commitment to strengthening applied epidemiology capacity and removing barriers to coordinated public health response. In 2006, a dedicated training unit was established within PHAC public health field services to provide the formal instructional component of CFEP and support broader workforce development. This unit, formalized in 2010 and now known as the Training and Development Unit (TDU), became responsible for delivering structured classroom training required under global FETP standards and accreditation (3), while also supporting ongoing professional development for field and adjacent staff.

The 2009 H1N1 pandemic further reinforced the need for scalable training models (4), improved technical fluency to interpret and appraise data (5), and enhanced coordination across jurisdictions. The event also increased interest in training related to vaccinology, vaccine hesitancy, and the role of vaccine campaigns in outbreaks. The TDU adapted its FETP training to suit, introducing new vaccinology training to its repertoire, and bolstering EIA case studies to include more focus on person-to-person disease outbreaks. Training on statistical software was introduced, focusing on data management, spatial epidemiology, and time series analysis. The TDU expanded its role to fill gaps and strengthen capacities as a result of non-pandemic emergencies as well. For example, training on cultural competency and mass gathering event surveillance was developed rapidly to support the public health needs of a large influx of Syrian refugees in 2016. This training was adapted in subsequent years to support asylum seekers at the Canadian border and refugee centres.

## Results

Lessons learned from earlier emergencies were magnified during the COVID-19 pandemic, which accelerated transformation in both scope and modality of applied epidemiology training (**Table 1**). As the public health workforce adapted to decentralized working conditions, the TDU also pivoted training to enable a wider reach and improved use of resources. The necessity for timely online training for specific tasks also became evident. For example, a gap in training for methods in contact tracing was identified, leading to a collaborative effort between the TDU and Health Canada to rapidly develop and deliver a new virtual course on this subject. While earlier training models required travel, adapting select courses to the virtual environment enabled increased capacity building among less resourced or remote health units, created greater opportunities to build networks across jurisdictions, and changed the resources required to operate the program (e.g., more varied skillsets for course development, but fewer in-person facilitators for training delivery).

**Table 1:** Timeline of events affecting field epidemiology training

Year	Milestone
1973	Epidemiology in Action (EIA) training developed
1975	Canadian Field Epidemiology Program (CFEP) formally established
2003	SARS pandemic
2004	Creation of the Public Health Agency of Canada (PHAC)
2006	CFEP sister programs launched: Field Services Training Unit and Canadian Public Health Service (CPHS)
2010	Field Services Training Unit launched as a formal independent entity to support CFEP and CPHS
2014	Just-in-time training for the 2014–2016 Ebola outbreak
2019	Field Services Training Unit renamed to Training and Development Unit (TDU)
2020	Just-in-time training on contact tracing for COVID-19. The EIA is adapted for virtual delivery and content tailored to the COVID-19 era
2021	Launch of public-health-focused R training and training on 2SLGBTQI+ epidemiology (both part of the core curriculum)
2022	Launch of Emergency Management and Public Health online training, TDU’s first mandatory training for all PHAC employees
2024	Launch of the TDU Brunch and Learn/Know Before You Go learning series, providing just-in-time training on emerging issues in public health in Canada and globally Collaboration with Training Programs in Epidemiology and Public Health Interventions Network (TEPHINET) to make scientific manuscript writing modules available in French
2025	Launch of training (core curriculum) on race-based data

Abbreviations: CFEP, Canadian Field Epidemiology Program; CPHS, Canadian Public Health Service; EIA, Epidemiology in Action; SARS, severe acute respiratory syndrome; TDU, Training and Development Unit; TEPHINET, Training Programs in Epidemiology and Public Health Interventions Network; 2SLGBTQI+, Two-Spirit, lesbian, gay, bisexual, transgender, queer, intersex, + other sexual and gender diverse communities

In 2019, the TDU broadened its mandate to include emergency preparedness training for federal public health staff in Canada, coinciding with the start of the COVID-19 pandemic. The TDU developed tiered, self-directed online learning for the Public Health Emergency Management (PHEM) curriculum, to enable learners to select the level of specialty required for their anticipated roles in an emergency response. This pivot toward a broad and inclusive approach to emergency management in Canada underscored that emergency management literacy was no longer exclusively the domain of specialized field responders. Public health professionals across disciplines require working knowledge of incident management systems, situational awareness reporting, risk communication, and operational coordination. As part of this new approach, foundational PHEM training is now mandatory for all staff at PHAC, and CFEP trainees must complete additional specialized training in this stream.

## Discussion

A modern approach to field epidemiology training: Embedding equity, climate, and cultural humility

Each year, the Chief Public Health Officer (CPHO) of Canada releases a report on the state of public health in Canada. These annual reports highlight current evidence on priority public health topics and provide aspirational guidance for the development of public health programming. For example, a recurring theme in recent CPHO reports has been health equity, which also became a key focus of the revised *Core Competencies for Public Health in Canada* (6–11). In recent years, CFEP training has therefore included offerings on race-based data for public health and applied learning on the Two-Spirit, lesbian, gay, bisexual, transgender, queer, intersex, + other sexual and gender diverse communities (2SLGBTQI+) epidemiology to support the collection and interpretation of disaggregated data, the application of equity lenses in surveillance and outbreak investigations, and culturally responsive engagement.

## Limitations

Technological fluency has become paramount in public health and field analyses, prompting major shifts in the Canadian FETP training landscape. For example, over the past decade, R software has become one of the most popular open-source data-languages in public health and epidemiology, enabling powerful analytical methods, professional data visualizations, and scalable database management across public health units (12,13). Training in R has therefore become a core component of the CFEP curriculum and has attracted many trainees from public health jurisdictions across Canada. The TDU’s approach to R training includes topical case studies and examples that emphasize responsible data governance and stewardship, reproducible workflows, and the importance of ensuring analysts adopt ‘do-no-harm’ approaches when sharing and publishing results.

## Conclusion

### Looking forward: A vision for the future of field epidemiology training in Canada

As outlined above, the evolution of federal applied epidemiology training in Canada has historically been shaped by reactions to public health crises. For example, the SARS epidemic prompted institutional reform, resulting in the creation of PHAC. The response to H1N1 emphasized operational scalability, and coincided with the formation of a formal and dedicated training unit. Finally, the COVID-19 pandemic accelerated digital transformation and broadened emergency management training beyond field staff. Future preparedness requires moving beyond reactive adaptation, toward anticipatory capacity building. Training programs must prepare public health employees not only for known threats but for uncertain and intersecting challenges, including climate instability, misinformation and disinformation, demographic shifts, and widening health inequities. This requires cultivating a new generation of field epidemiologists with strong technical and domain expertise who are also empathetic, aware, and equipped with the interpersonal skills needed to enable systems thinking and work effectively across sectors.

In an era where social media influence has the potential for great monetary or political gain, misinformation and disinformation have become some of the most serious risks to public health (14,15). Public health relies on accurate and timely dissemination of information to educate the public about ways to avoid or mitigate health risks. Artificial intelligence has the potential to amplify misinformation and disinformation, but it also presents new opportunities across several aspects of field epidemiology. Training the next generations of field epidemiologists to optimize evolving technologies, while recognizing their limitations and critically thinking about results, will be an ongoing, but important challenge.

Training modalities will continue to adapt to the accessibility needs of the public health workforce, accommodating non-traditional working environments and supporting a variety of learning needs and preferences. For example, courses that were once delivered as week-long intensive sessions may be broken down into smaller modules delivered over longer time periods, interspersed with intersession activities to apply the knowledge learned. A variety of mediums will continue to be explored, including recorded videos, collaborative work, case studies, webinars, discussion panels, podcasts, and online synchronous and asynchronous platforms. Training options, such as complex simulations, online gaming modalities, and virtual reality exist, but they must be assessed for accessibility before implementation.

Field-based learning must remain central, not only to strengthen operational readiness but to cultivate cross-jurisdictional relationships at the responder level. At the same time, training must intentionally build the capacity to recognize whose voices and perspectives are missing and to facilitate inclusion before an emergency occurs. By continuously adapting training to public health realities, the program aims to encourage future generations of CFEP trainees to pair scientific and operational excellence with empathic, human-centred responses.
